# A magnetic resonance imaging study on the articulatory and acoustic speech parameters of Malay vowels

**DOI:** 10.1186/1475-925X-13-103

**Published:** 2014-07-25

**Authors:** Alireza Zourmand, Seyed Mostafa Mirhassani, Hua-Nong Ting, Shaik Ismail Bux, Kwan Hoong Ng, Mehmet Bilgen, Mohd Amin Jalaludin

**Affiliations:** 1 Biomedical Engineering Department, Faculty of Engineering, University of Malaya, Kuala Lumpur, Malaysia; 2 University Malaya Research Imaging Center and Department of Biomedical Imaging, University of Malaya, Kuala Lumpur, Malaysia; 3 Radiology Department, Faculty of Medicine, Erciyes University, 38039 Kayseri, Turkey; 4 Department of Otorhinolaringology, Faculty of Medicine, University of Malaya, Kuala Lumpur, Malaysia

**Keywords:** Vocal tract shape, Articulators’ movements, Malay vowel sounds, Active contour, Acoustic parameters, Formant frequencies

## Abstract

The phonetic properties of six Malay vowels are investigated using magnetic resonance imaging (MRI) to visualize the vocal tract in order to obtain dynamic articulatory parameters during speech production. To resolve image blurring due to the tongue movement during the scanning process, a method based on active contour extraction is used to track tongue contours. The proposed method efficiently tracks tongue contours despite the partial blurring of MRI images. Consequently, the articulatory parameters that are effectively measured as tongue movement is observed, and the specific shape of the tongue and its position for all six uttered Malay vowels are determined.

Speech rehabilitation procedure demands some kind of visual perceivable prototype of speech articulation. To investigate the validity of the measured articulatory parameters based on acoustic theory of speech production, an acoustic analysis based on the uttered vowels by subjects has been performed. As the acoustic speech and articulatory parameters of uttered speech were examined, a correlation between formant frequencies and articulatory parameters was observed. The experiments reported a positive correlation between the constriction location of the tongue body and the first formant frequency, as well as a negative correlation between the constriction location of the tongue tip and the second formant frequency. The results demonstrate that the proposed method is an effective tool for the dynamic study of speech production.

## Introduction

The investigation of articulator shape during speech production can facilitate the understanding of the mechanisms of speech production. According to the acoustical theory of speech production [1], understanding speech production requires consideration of the vocal tract as an acoustical tube as its cross-sectional area changes during the speech production process [2]. Various studies were performed to support this theory when it was first suggested. From the 1940s to the 1970s, a large number of radiography experiments were conducted to collect the data that revealed the shape of the vocal tract during speech production. For subsequent research on acoustic speech production, the collected data were employed to develop early analog models for articulation. In the succeeding decades, continued research coupled with the advent of computers resulted in remarkable advancements in modeling the articulatory and acoustic processes. In addition, articulation models have been used to study the more complex aspects of modeling, such as the three-dimensional shape of the tongue and its movements [3-10].

Different instruments have been used by researchers to measure the shapes of the vocal tract and articulators. The X-ray CT method is a powerful tool for this purpose. As a considerable part of the entire vocal tract length that is observed by X-ray CT imaging, 3D information that indicates the shape of the vocal tract, as well as tongue shape and movement pattern, is obtainable [11]. Nevertheless, this method presents certain drawbacks that decrease the use of such a system including the harmful effects on humans in relation to the X-ray imaging instrument. Dynamic data on tongue movement in the oral cavity can be provided by an X-ray micro beam and electromagnetic articulography [12,13], which are categorized as point tracking tools. Ultrasound scanners can supply dynamic images of moving structures in the oral cavity, such as the tongue surface in both midsagittal and transverse planes [14,15]. Nevertheless, ultrasound transmission properties limit the use of such devices to mapping anterior airway surfaces.

The disadvantages of using the aforementioned methods motivate us to employ the MRI system in this study. One of the most significant points concerning MRI for non-medical purposes is its ability to provide images similar to those obtained by X-ray CT but without any side effects from radiation. MRI is unconstrained by the positioning of a subject in obtaining images of different directions and angles. Images of each slice of the vocal tract are obtainable with an acceptable quality for speech production study.

Many researches have been done on dynamic or static study of vocal tract based on MRI. Technology development in magnetic resonance imaging has made investigation of articulators during speech production feasible. Real-time MRI for speech production has been studied in different languages [16-18] such as French [19], German [20], Swedish [21], European Portuguese [22,23], Finnish [24], Czech [25] and Japanese [26]. In Malay language, however, no research has been performed on this matter. Here, dynamic study of prolonged Malay vowels is performed. Investigating the production of Malay vowels would be helpful in diagnosing articulation disorders. In particular, the data such as this could be useful as a standard vowel pronunciation of normal people which can be compared with other data to determine any disorder in this matter. K-space in acquisition techniques including partial Fourier or spiral acquisition method is frequently used for increasing the temporal MR resolution [16,27,28]. Information provided by different vocal tract measurement techniques has been used in developing some kinds of biomechanical simulation tools for simulating the movements of the muscles in vocal tract [29,30]. The simulation tool [31] has been employed in some further studies to determine the functions of vocal tract organs [32-35].

However blurring of some parts of the acquired image is still a drawback for this technology because during the scanning time the subject needs to remain to be stationary (see [36,37] for the challenges in MRI study of articulation). As a remedy for the blurring problem, in some studies, a stroboscopic method is employed to recapture some images for the same speech in different periods in order to produce a reliable MR sequence [38]. However, some limitations for this method are apparent. For example, the speaker needs to repeat the utterances several times. Not all mistakes by the speakers can be avoided since exact repetition is not possible. In other words, a main bottleneck for this research is that many effective factors during articulation change from one speaker to another, which is referred to as interspeaker variability [39]. This variability can be categorized as anatomical and psychological features [28,40-42]. In addition, in Malay language no study of speech production based on dynamic MRI has been done so far. Consequently, this study is considered a pioneer in the framework of the dynamic study of speech articulation in Malay language based on MRI.

MRI however presents certain disadvantages, such as the duration of the scanning process. Sometimes scanning takes several minutes, which can be tedious for subject. In the study of the pronunciation of phonemes, the subject is required to utter the speech sound several times [43], which can be strenuous. Additionally, because of partial blurring, the images obtained by MRI are sometimes of unacceptable quality. Another drawback of MRI is the low image contrast between tissues with low hydrogen content and airways. Consequently, segmenting the scanned image to determine the regions occupied by airways (such as the oral cavity) can result in errors [44]. In MRI, the quality of the object in an image depends on the thickness of the scanned tissue. Usually, MRI provides clear and undistorted images from the object with the thickness of at least 3 mm. Moreover, the loud sound produced by the gradient coils during scanning interferes with the voice of subject during the recording process. Despite these drawbacks, an MRI system provides valuable information on the vocal tract shape that is formed as subject’s uttered speech. To address the image-blurring problem during the scanning process, this study proposes image processing techniques including active contours for the use of MRI in studying articulation. The results indicate that these techniques enable the measurement of articulation parameters efficiently.

Research was previously conducted using a 3D reconstruction of the vocal tract (from MR images) for speech simulation [27]. The study employed the region growing method to obtain axial slices from the vocal tract. However, as slices of the vocal tract are obtained, the tongue performs several partial movements as the subject pronounces a phoneme and it is difficult to stay absolutely still for a prolonged time. Consequently, scanned images of certain regions on the tongue boundaries may be of insufficient quality given that even minor tongue movement blurs the scanned images. Thus, the accuracy of evaluating the vocal tract slices by region growing techniques decreases. As a remedy, researchers have suggested the use of human operators to trace the boundaries of the oral cavity and region growing methods that require the determination of the initial seeds in the growing regions [45]. Most of the relevant methods mentioned in literature [22,40,43,46-48] are semi-automatic and consequently require human intervention, making the process tiresome for specialists, and therefore, prone to error. In this paper, we employ an active contour that focuses on the tongue tracking. By determining the number of control points of the active contour with an automated method, we control its degree of freedom, thereby enabling a smooth and relatively accurate evaluation of the tongue boundary even when this boundary is partially blurred in MR images.

Active contours, or “snakes”, are mathematical models that define deformable curves on the image domain. These methods, categorized as deformable models, are of special interest for medical image segmentation [23,47,49]. In this framework, internal and external forces influence the deformation of the curves. Internal forces are dynamically defined and computed from the curve characteristics, and external forces are obtained mostly from the image in which the active contour is applied.

According to the literature, active contours are divided into two categories: geometric [50-55] and parametric active contours [56,57]. Kass et al. in 1987 were the first to attempt the development of an active contour based on the energy minimization of splines and external constraints, including the energies defined by the image edges that deform curves. To smoothen the curves, the authors defined an internal energy based on curvature. However, the weak points of their active contour model, including sensitivity in the selection of initial points and its inability to track non-convex objects, motivated modifications to their model.

Williams and Shah in [57] introduced the greedy snake algorithm. They employed a fully discrete method to compute the movement of the snake. For this purpose, the neighborhood pixels of each snake point were used to identify the minimum energy obtainable for the movement. Furthermore, an efficient method for evaluating the curvature of discrete curves was employed.

In our experiments to investigate tongue shape and movement, the materials we considered include the pronunciation of a preselected set of Malay vowels. To this end, our subjects were made to lie on an MRI scanner were asked to pronounce the Malay vowels. The mouth region of the head, including the oral cavity, tongue, and lips, was examined during the experiments. The active contour employed in this approach required tracking the tongue in the MRI frames. To prevent lengthy computations of more sophisticated active contour algorithms, the greedy active contour model was employed. Image preprocessing techniques including morphological filtering were applied to MR images to ensure effective performance despite partial image blurring.

## Methods

### MRI scanning parameters and image acquisition protocol

Medical ethic approval was obtained from University of Malaya Medical Center (UMMC) before conducting the experiments. The MR images for this study were obtained using a General Electric SignaHDX 1.5 Tesla scanner. T1-weighted sagittal MRI data on two subjects (one male and one female) for six different Malay vowels were acquired using the imaging protocol described in Table 1 and anatomical information of subjects are summarized in Table 2. Such information can help readers to compare the data in the current work with other data sets. Moreover, information on the physical dimensions of a subject enables clear envisioning of an individual’s body structure. The scanning protocol employed in this study was adopted to pre-synchronization technique which automatically triggered the scanner based on heartbeat of the subject [43]. Meanwhile the subjects used headphones to listen to the operator’s commands and their heartbeats for synchronizing their articulation with their measured cardiac. The subject started their speech after receiving the command from the operator and at the same time of hearing their heartbeat. They continued to articulation until hearing their 6th heartbeat. Then they inhaled and waited for the next command from the operator. This procedure was repeated for 6 times to ensure having enough MRI frames. As the triggering was performed based on the heartbeats of the subjects and the subjects attempted to make their utterances synchronic with their heartbeats, the utterances were synchronized with the scanning process. Consequently, MRI frames from several periods of articulation were provided from each vowel.

**Table 1:** MR parameters for vocal tract image acquisition

**MODE**	**MR Echo, using 8 channel cardiac coil**
TE	4.5 ms
TR	65 ms
ETL (echo train length)	18ms
Flip angle	70 degree
FOV	36 cm
Matrix	256 × 256 pixels
Resolution	1.057 pixels/mm
Slice thickness	7 mm

**Table 2:** Anatomical information of subjects

	**Subject**	
	**Male**	**Female**
Age	27	25
Height	167 cm	151 cm
Weight	64 Kg	52 Kg
Head circumference	56.5 cm	55.5 cm
Neck circumference	32 cm	30 cm

To reduce image blurring during image acquisition, the subjects were required to maintain vocal tract shape (i.e., hold the mouth position constant for a certain period) as they pronounced the vowels. Prior to the scanning, the subjects performed phonation practice. Some assumptions were made on the basis of a scanning protocol, described as follows. To reduce the intensity of the sounds heard by the subjects during the imaging process, the subjects were asked to use earplugs.

Afterwards, they were positioned on the MRI table in a comfortable state. Pieces of cloth were placed under their heads to limit their head movement to a minimum. We positioned the heads of the subjects in the center of the magnet. As the experimental condition that must be taken to the consideration is the head, particularly the upper jaw of the subject, it should not move during the experiments. Prior to each image acquisition session, a sagittal localizer was used to provide an appropriate field of view for the scanning location. Subject utterances during the scanning were recorded but due to the noise of the environment, the recordings were not reliable.

### Speech corpus

To conduct a dynamic study of vowel production, we asked the subjects to pronounce several repetitions of six prolonged Malay vowel sequences (/a/, /e/, /ә/, /i/, /o/ and /u/) during the scanning process. In addition to the MRI scanning process, for acoustical analysis of the speeches, the subjects were asked to pronounce the same Malay vowels for 5 s each at a comfortable pitch and loudness level. The speech sounds were recorded using a Shure SM58 microphone in a regular room environment. The mouth-to-microphone distance was fixed at 2–3 cm. Gold-Wave digital audio editor software was used to record the speech sounds at a sampling rate of 20 kHz with 16-bit resolution. There was no co-articulation either in the recording speech nor in MRI scanning process. To date, no dynamic MRI-based study has been performed on the production of prolong Malay vowels.

### Formant frequencies extraction

Besides MRI data for the study of the articulatory parameters, the Praat software was used to determine formant frequencies of the prolonged vowels of the subjects [58] based on the recordings. The following standard formant settings were used: 5500 Hz of maximum formant frequency for female and 5000 Hz for male subjects, five formants, 25 milliseconds of window length, and a dynamic range of 30 dB. There were two possibilities for extracting formant frequencies using Praat, namely, Praat manual extraction as well as the extraction of automatic formant frequencies using Praat scripting. In this study, the formant frequencies were obtained using the automatic method, and the average values were used instead of the middle point value; this decreased the possible error of the Praat calculation of formants because instead of one point for each sample, several points were extracted from each sample and then the average was calculated. The number of points used for each sample depended on the sample length, and it was equal to the length of the sample divided by the length of the window frame (25 milliseconds).

### Instrumentation and data collection

In a large number of MRI studies [27,43,45,59], authors dismiss the focus on the contour extraction of MRI frames. The reason can be an implicit assumption that high-resolution MR images with acceptable contrast and quality are collected. Consequently, image processing software extracts contours for the quantitative investigation of articulatory parameters. In general, however, this supposition does not hold. As the tongue moves during imaging, blurring is unavoidable. Under these circumstances, the extraction of tongue contours in advance is a challenging task.

Numerous methods are used to enhance acquisition of MR image sequences and appropriate trigger systems have been proposed. In clinical practice, however, the triggering method based on electrocardiogram monitoring is performed in some studies [43,59]. To increase the temporal resolution for real-time imaging, researchers put forward some other techniques [16,27,28]. In these methods, images are acquired at different speeds on the basis of ultrafast imaging sequences. Multiple echoes during the imaging process are employed. However, because of partial motion of subject during scanning process motion artifacts are observed in the yielded images.

To resolve blurring in MR images, we propose an active contour-based method for extracting tongue contours in MRI frames. By determining the control points of active contours, the tongue contours can be traced even when the tongue is partially blurred. If the blurring is not severe, the traced contours are reliable for the experiments. Otherwise, the blurred frames are ignored and other frames are used for analysis.

### Active contour

Kass et al. [56] were the first to develop a framework in which a deformable snake moves toward an object as a result of constraint forces imposed via an energy minimization strategy. The term “snake” arises from the way that the active contour moves to minimize energy. By applying some modifications to the active contour model of Kass et al. including the use of a fully discrete method for the snake movement, Williams and Shah [57] created the greedy snake algorithm. In this model, for each point located in the neighborhood of a snake control point *v*(*s*_*i*_), three energy terms were computed. Afterwards, the combined energy was obtained by the summation of the three energies as follows:

(1) $$E_{\mathrm{comb}}\left(x,y\right)=\alpha \left(s_{i}\right)E_{\mathrm{ela}}\left(x,y\right)+\beta \left(s_{i}\right)E_{\mathrm{curv}}\left(x,y\right)+\gamma \left(s_{i}\right)E_{\mathrm{img}}\left(x,y\right)$$

where *E*_*ela*_(*x*, *y*) denotes the elasticity energy, *E*_*curv*_(*x*, *y*) stands for the curvature energy, *E*_*img*_(*x*, *y*) is the image energy, and (*x*, *y*) are the indices to the pixels in the neighborhood, where

(2) $$v\left(s\right)=\left[\begin{array}{c} x\left(s\right)\\ y\left(s\right) \end{array}\right]$$

The elasticity energy is obtainable by the following formula:

(3) $$E_{\mathrm{ela}}\left(x,y\right)=\bar{d}-∥v\left(s_{i}\right)-v\left(s_{i-1}\right)∥=\bar{d}-\sqrt{{\left(x\left(s_{i}\right)-x\left(s_{i-1}\right)\right)}^{2}+{\left(y\left(s_{i}\right)-y\left(s_{i-1}\right)\right)}^{2}}$$

Where $\bar{d}$ denotes the average distance between all the points in the snake. The curvature energy for the neighborhood is

(4) $${∥v\left(s_{i+1}\right)-2v\left(s_{i}\right)+v\left(s_{i-1}\right)∥}^{2}$$

The last term in Equation (1), *E*_*img*_(*x*, *y*), indicates the effect of energy on the processed image; this energy forces the snake points to be attracted to the object of interest. This term is computed thus:

(5) $$E_{\mathrm{img}}=-{∥\nabla \left[G_{\sigma }\left(x,y\right)*I\left(x,y\right)\right]∥}^{2}$$

where *G*_*σ*_(*x*, *y*) stands for a two-dimensional Gaussian blurring filter with a standard deviation of *σ*. The filter is used to blur the image gradient, thereby influencing the snake by the image gradient from a larger distance.

Finally, the stopping criterion for the snake movement depends on the minimum number of points moving in each stage, as well as the maximum number of iterations allowed for the snake. The stopping criterion is given as follows:

(6) $$\frac{∥v{\left(s\right)}^{t}-v{\left(s\right)}^{t-1}∥}{n}<th_{\mathrm{stop}}$$

where vector *v*(*s*)^*t*^ contains the indices to the snake points at time step *t* and *v*(*s*)^*t* − 1^ contains the snake points at time step *t* - 1. *n* and *th*_*stop*_ denote the total number of control points in the snake and a threshold for the stopping criterion [60].

### Tongue properties from an articulatory perspective

As mentioned, the upper boundary of the tongue is critical for producing vowel sounds. As a result, the active contour aims at tracking the upper boundary of the tongue. For this purpose, some preprocessing steps such as dilation and erosion operations are performed to obtain the initial points for the active contour. The initial points of the active contour employed in this study are divided into two groups: upper initial points and lower initial points.

### Obtaining initial points for the active contour

**Figure 1:**
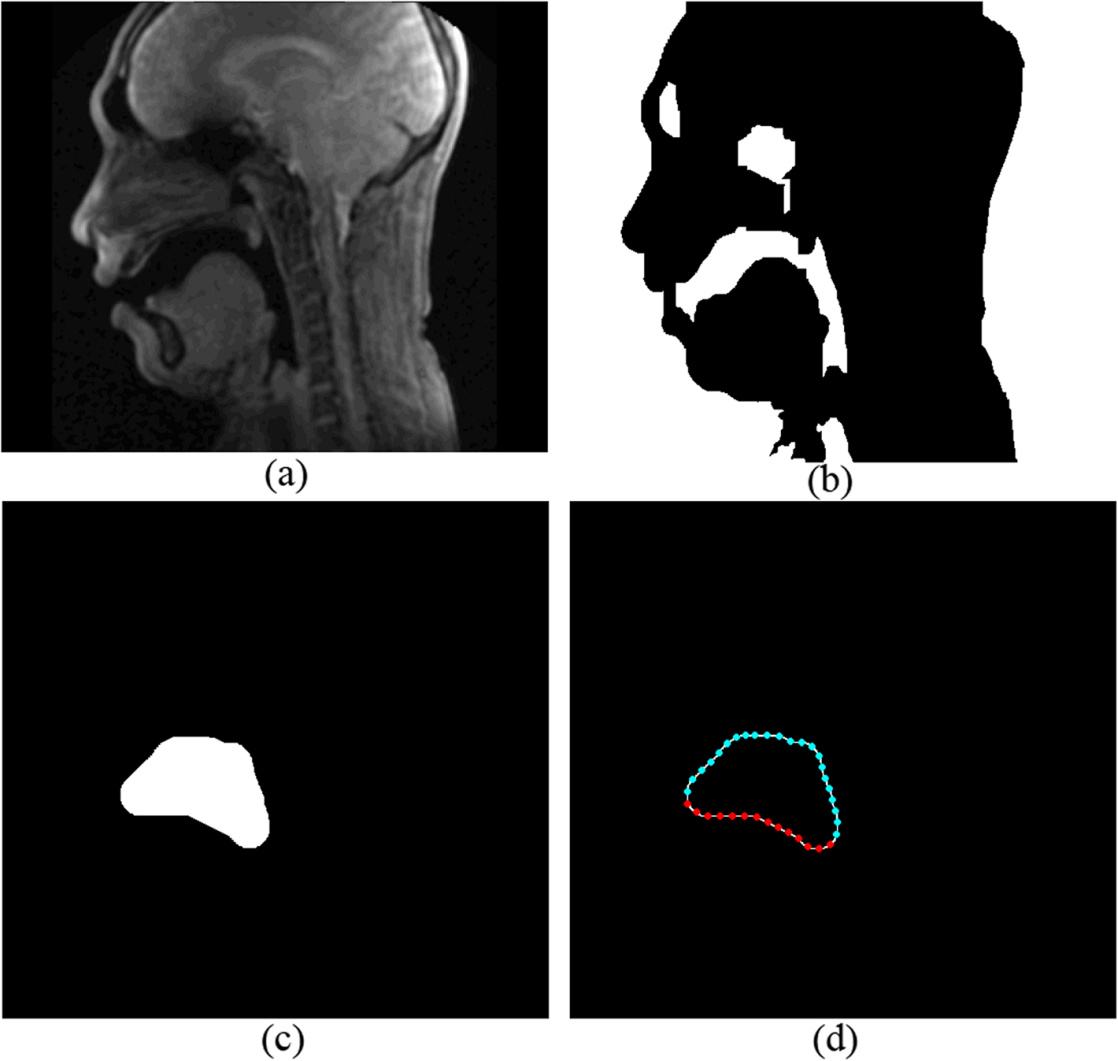
(a) One of the MRI frames of male subject investigated in this study; (b) the cavities obtained after the preprocessing operations; (c) a shape obtained from the oral cavity to provide the initial points of the active contour; and (d) the initial points of the active contour (lower in red and upper in yellow).

The upper initial points are obtained from the oral cavity because the tongue movements are restricted to the oral cavity region. Figure 1(a) presents one of the investigated MRI frames. Given that the initial points of the active contour are the same for all the frames, a frame containing the largest oral cavity is more appropriate for our purpose as it results in initial points with highest distance. Under this circumstance morphological operation can extract the oral cavity more efficiently and certainly morphological filtering does not remove a part of oral cavity as a redundant segment.

Oral cavities appear in MRI frames as dark regions; thus, by applying a threshold near the zero level, they are discriminated from the other parts of the images. Determining an appropriate value for the threshold can be accomplished by a human operator as well as by a histogram-based algorithm. The threshold determined for this step of preprocessing (*th*_*I*_) is obtained as follows:

(8) $$0.06.\left(IM_{\text{max}}-IM_{\text{min}}\right)+IM_{\text{min}}=th_{I}$$

**Figure 2:**
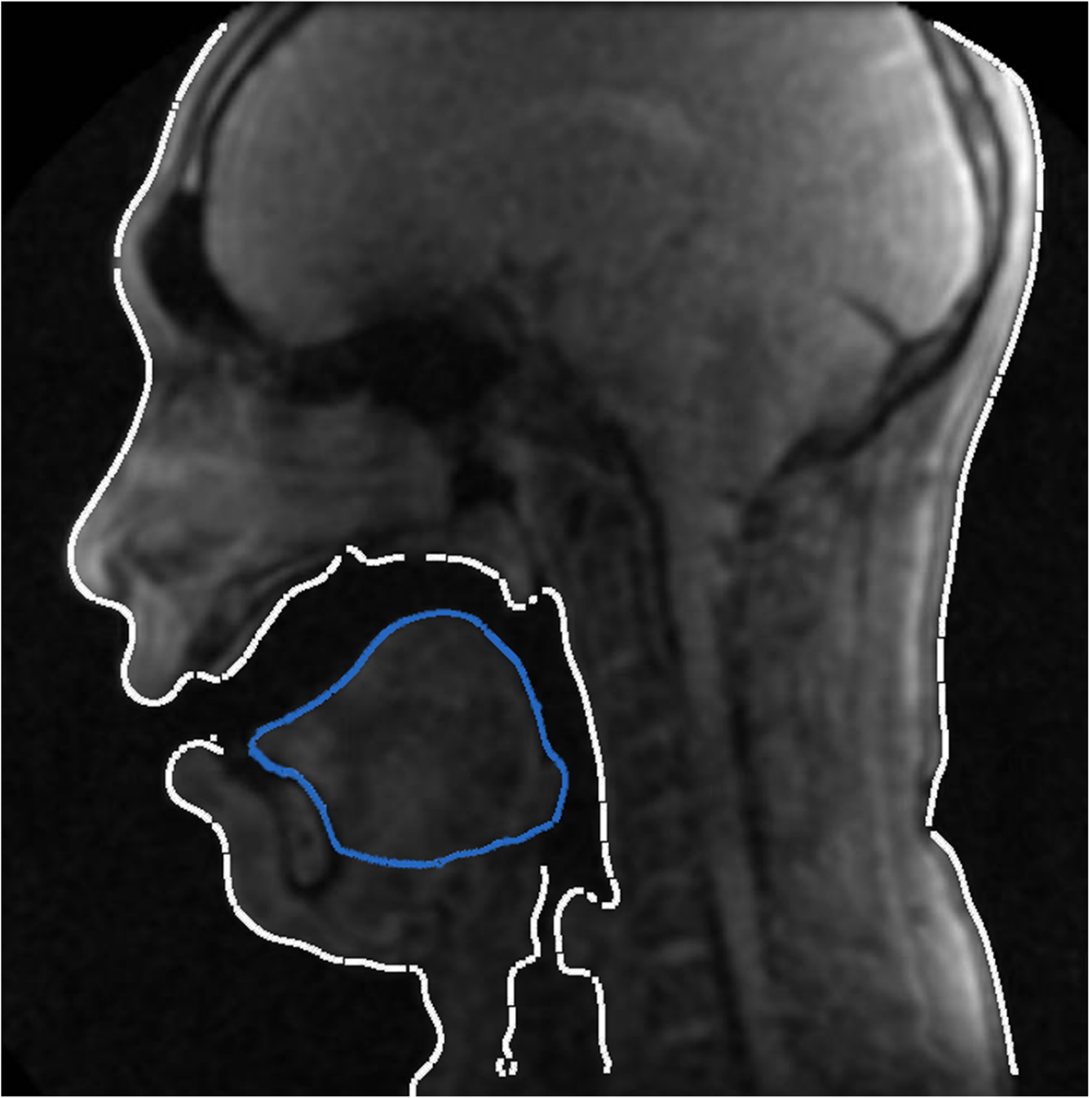
**A sample of MRI frame of male subject.** The contours provided by the proposed method. The blue contours have been provided from the active contour while the white contours obtained from preprocessing.

After discriminating the oral cavity, performing a number of morphological operations including opening operation with disc-shaped structure element can help provide a smooth area that is representative of the tongue location. The upper boundary of the oral cavity is also obtained in the preprocessing step.Figure 1(c) presents the part including the oral cavity and the upper part of the tongue, that are obtained from the morphological operations. The shape in Figure 1(c) has been obtained by applying the threshold on Figure 1(a), followed by applying the closing operation with a spherical structure element on the corresponding segment in Figure 1(b). The initial points of the active contour are selected from the boundary of the obtained shape as shown in Figure 1(d). The lower initial points of the active contour are immovable (fixed points) because lower boundary of the tongue are obtained by some preprocessing rather than using the active contour. As a result, the points are selected from the lower boundary of the shape obtained in the previous step.In addition to the contours of oral cavity region, other contours of the image are provided by applying threshold (computed by Equation 8) to the image, followed by segmentation using connected component operations and employing morphological filtering. In particular, after applying threshold, a binary image containing a large number of segments is obtained. Some of the redundant segments are filtered out based on their size and their proximity to the segment of the jaws. Dilation of the jaws segment with a certain size of a disc-shaped structure element can help in finding these segments. Following the removal of the redundant segments, contours of the segments are obtained by using morphological operations. In particular, a subtraction of dilated segments from their eroded version results in contours of the segments (Figure 2).

## Results

**Figure 3:**
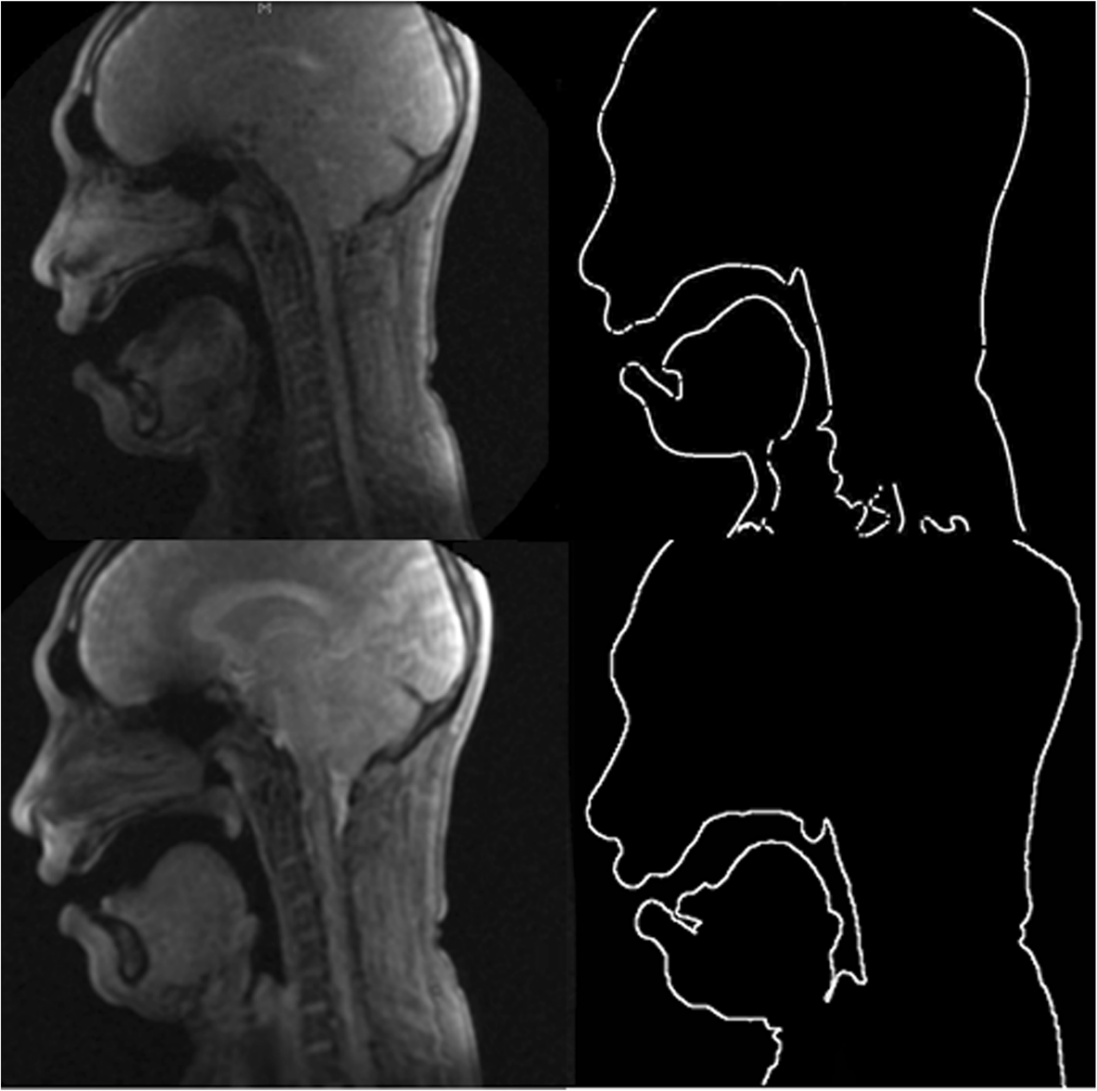
Samples of low and high contrast MR frames and the contours obtained by the proposed method.

**Figure 4:**
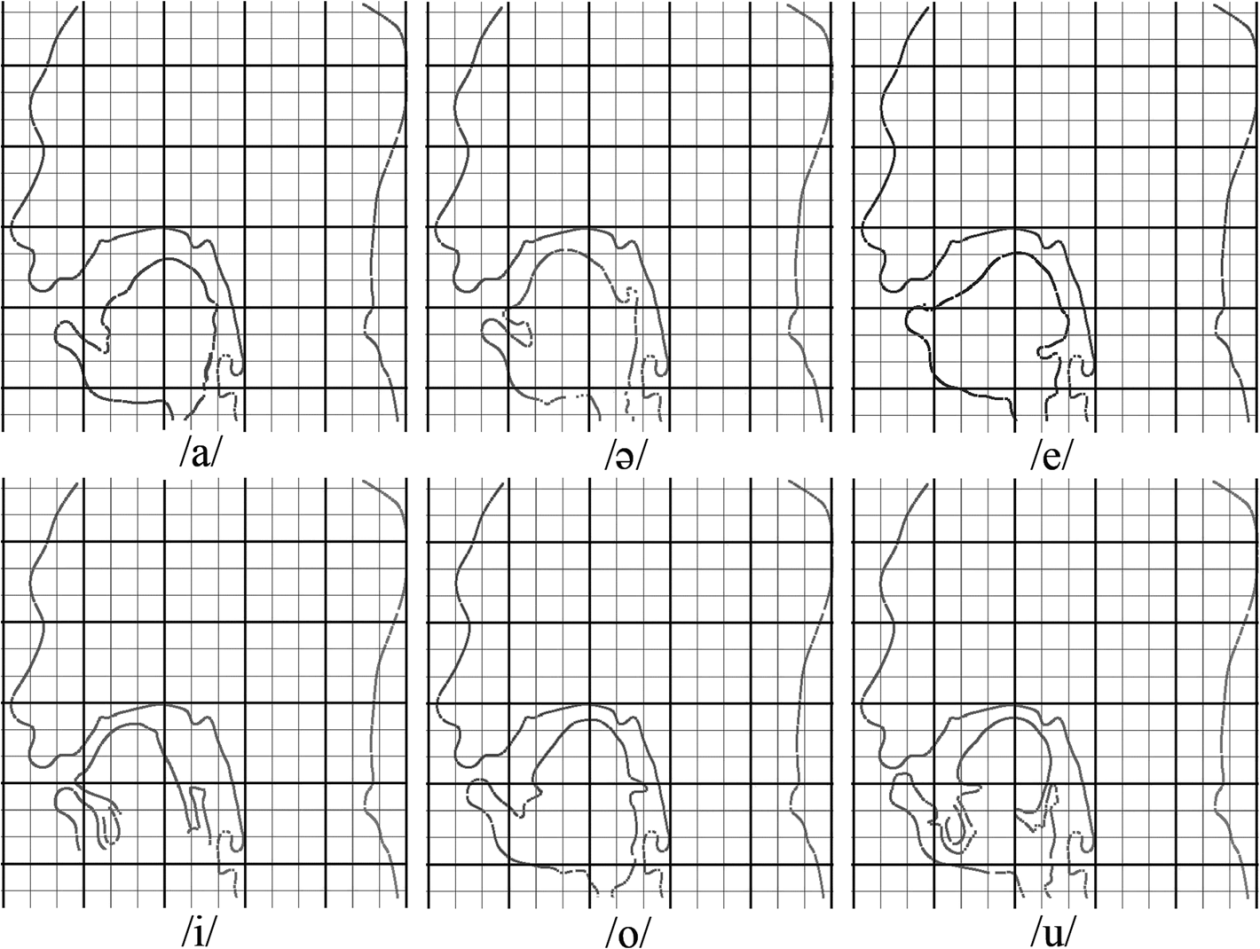
The tongue contours after the production of each vowel and positioning of the tongue in a steady state.

**Figure 5:**
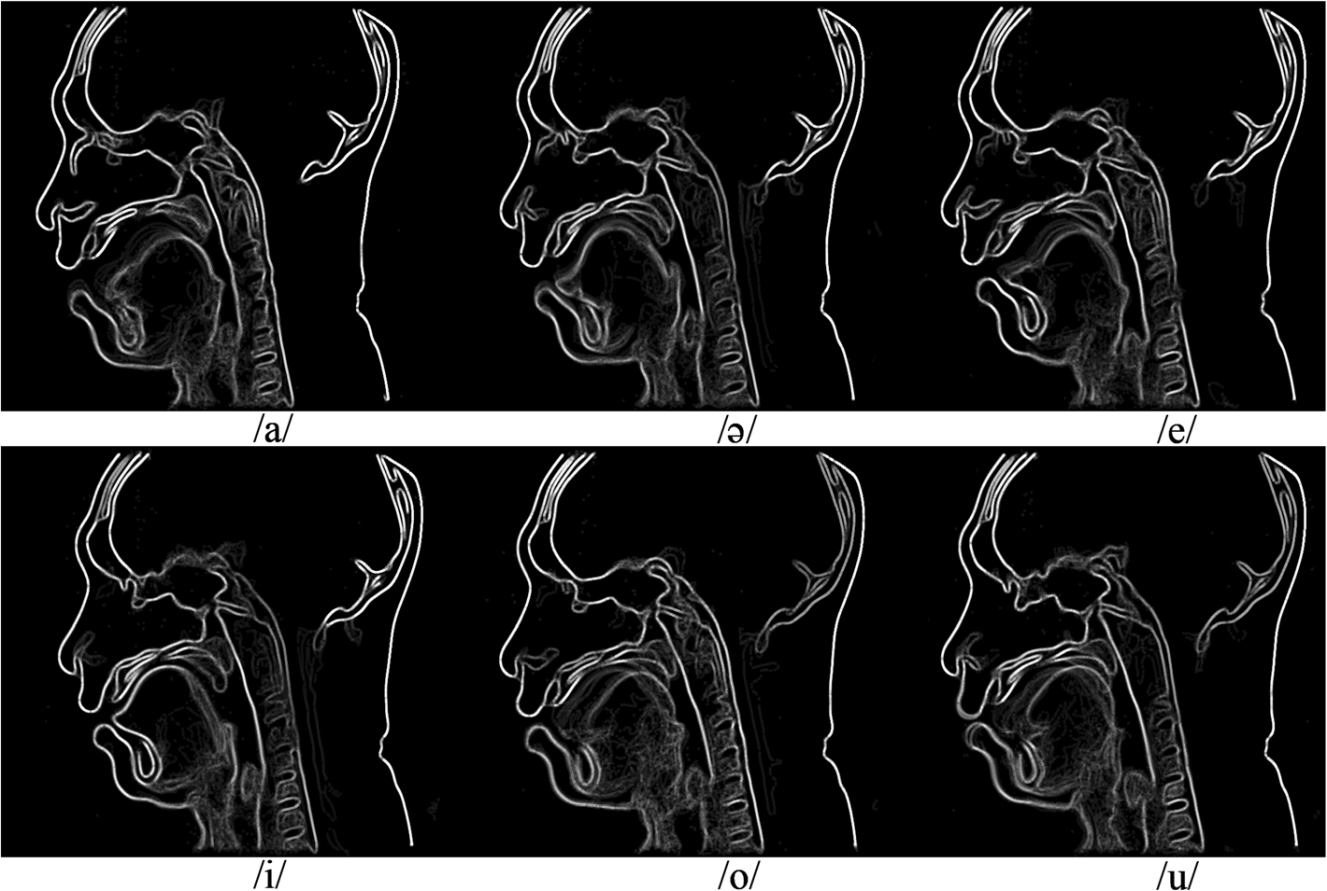
Tongue movement effect in different vowels.

Under the aforementioned protocols, a variable number of MR images were acquired from the subjects as they produced the vowel sounds. Therefore, the dynamic study of vowel production is feasible. The average frame rate obtained by this method was 5 frames/second. The resolution of each frame was 1.057 pixels/mm. The active contour parameters *α*, *β*, *γ*, *σ*, maximum iteration, and *th*_*stop*_ employed to obtain the contours were 1.2, 1 and 5.2, 5, 200, and $\frac{n}{10}$, respectively. The values of the active contour parameters were obtained by a manual trial and error experiment on one frame from a male subject. Afterward, these parameters were used for the whole of the experiments. According to the examples of acquired MR images, which are shown in Figure 3, a low contrast region of tongue can be extracted properly by using the proposed segmentation method.Figure 3 shows the tongue and oral cavity contours obtained by the proposed method. It is possible to measure quantitatively the articulatory parameters as shown in Figure 3. For the production of each vowel, the tongue moves to form the appropriate shape in the oral cavity. The shape of the tongue after its movement was observed and measured to create a baseline for articulation disorder studies. Figure 4 presents the plots of tongue contour coordination during the production of each vowel.To investigate the movement of the tongue during vowel articulation, the average of extracted contours for different frames was computed for each individual vowel. Figure 5 presents the effect of tongue movement during articulation of Malay vowels. The blurred parts represent the tongue contour movement while the bright white parts belong to unmovable contours.

At first glance it is observable that the highest area of oral cavity is obtainable while pronouncing vowel /a/. Comparing the tongue structure while pronouncing the vowel /a/ and /ә/ a tongue tip and tongue body raising in vowel /ә/ is observable. In contrast, a back raising of tongue is shown for articulation of vowel /e/ in comparison with /a/ and /ә/. Front raising of tongue in vowel /i/ is considerable. Tongue shape in vowel /o/ and /u/ is quite similar and both of the vowels show a considerable tongue back raising but the observable difference is the lip aperture which is higher in vowel /o/. As a summary back raising in vowels /o/, /u/, and /e/ are dominant while front raising in /i/ and /ә/ are dominant.

In addition to the tongue shape for each vowel, three common articulation parameters were measured [43,59]:

1. Tongue tip constriction location (TTCL),

2. Tongue body constriction location (TBCL), and

3. Lip aperture (LA; distance between the upper and lower lips).

The measurements are done with the coordinate system based on the palatal plane which is an anatomical standard plane in the midsagittal slice and can be drawn based on a line from the anterior nasal spine to the posterior nasal spine.

**Figure 6:**
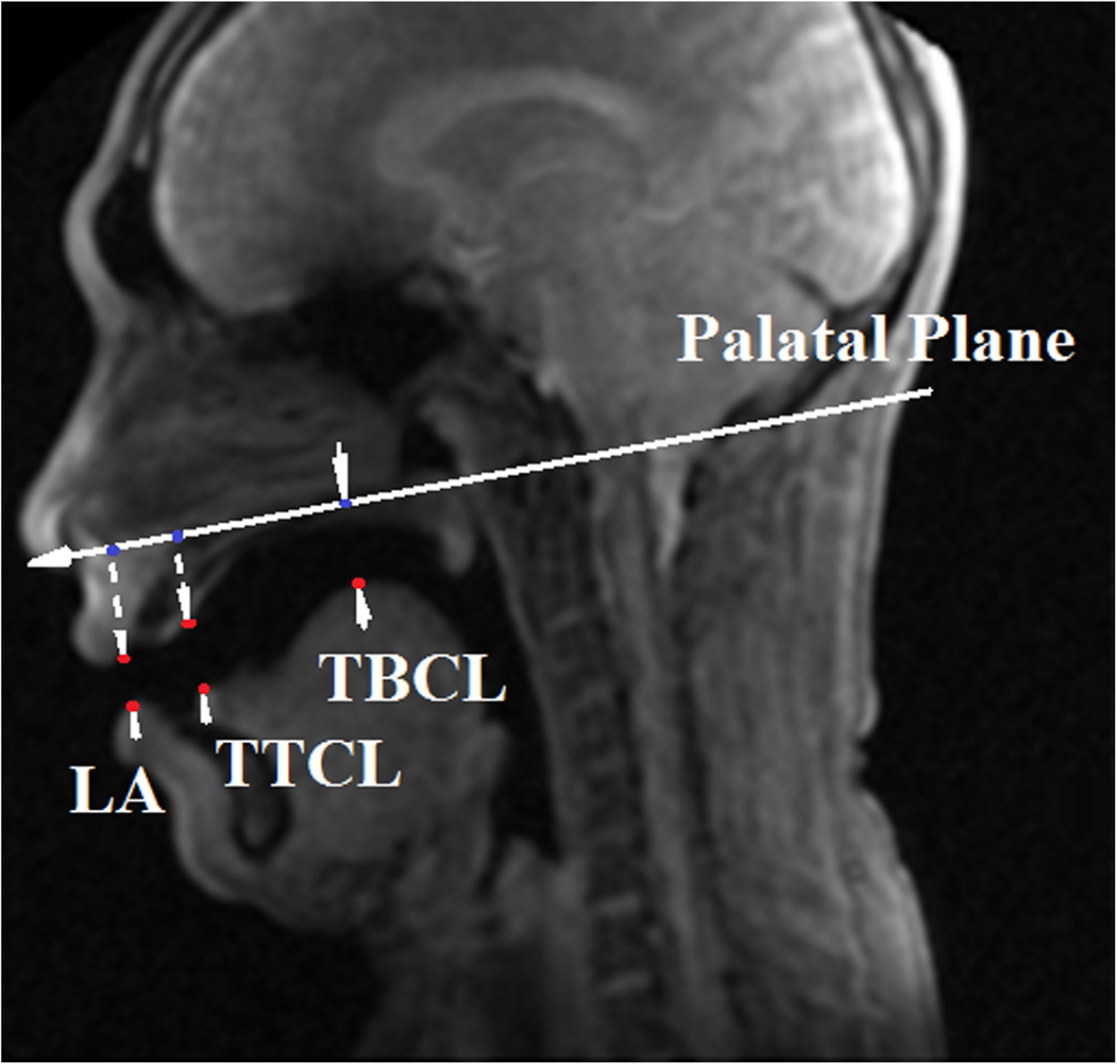
Understanding the articulatory parameters TTCL, TBCL, and LA.

Table 3 presents the speech articulation parameters measured in this study. To perform a comparative study of the tongue position for the articulation of different Malay vowels, the TTCL and TBCL were measured. Figure 6 shows the measurement of the articulatory parameters.

**Table 3:** Articulatory parameters obtained in this study

**Vowel**	**Gender**	**Articulatory parameter**	**Measurement (mm) of articulatory parameters by frame number**							**Mean**	**STD**	**Variance**
			**1**	**2**	**3**	**4**	**5**	**6**	**7**			
a	M	TTCL	19.47	21.57	25.25	28.67	27.88	24.73	20.78	24.05	3.5528	12.6226
		TBCL	9.47	13.42	11.84	12.63	5.26	12.1	10.26	10.7114	2.7593	7.6137
		LA	11.31	12.63	12.1	12.36	11.31	12.63	11.84	12.0257	0.5637	0.3178
	F	TTCL	17.01	25.42	27.7	30.5	29.1	27.35	24.37	25.9214	4.4411	19.7238
		TBCL	6.66	8.24	10.69	12.27	10.34	12.27	8.06	9.79	2.1835	4.7676
		LA	4.91	8.76	13.32	15.42	10.86	12.09	5.08	10.0629	4.0235	16.1885
e	M	TTCL	11.57	12.63	13.68	14.73	16.05	14.47	13.15	13.7543	1.4799	2.1901
		TBCL	8.21	9.79	8.47	11.36	11.1	7.94	10.05	9.56	1.3868	1.9233
		LA	9.73	11.57	11.31	11.05	13.42	12.1	12.1	11.6114	1.1325	1.2826
	F	TTCL	13.6	19.03	21.13	23.23	27.09	22.88	20.78	21.1057	4.1678	17.3706
		TBCL	7.23	6.88	7.75	8.63	8.98	7.75	8.63	7.9786	0.7877	0.6205
		LA	1.58	5.08	10.34	11.74	13.84	12.97	12.97	9.7886	4.6605	21.7201
ә	M	TTCL	12.31	15.47	19.68	21.52	25.47	23.63	20.73	19.83	4.5734	20.9164
		TBCL	6.09	4.51	7.93	9.25	10.56	9.25	11.88	8.4957	2.5445	6.4743
		LA	6.58	6.84	6.58	8.15	7.1	7.89	9.47	7.5157	1.06	1.1237
	F	TTCL	13.67	21.38	23.48	25.76	30.67	25.06	22.6	23.2314	5.1701	26.7298
		TBCL	3.33	5.96	5.78	5.96	7.01	5.96	6.13	5.7329	1.1343	1.2866
		LA	3.86	11.74	10.34	8.76	12.27	7.71	10.51	9.3129	2.879	8.2887
i	M	TTCL	6.31	5.52	6.05	6.05	6.84	6.84	8.15	6.5371	0.851	0.7243
		TBCL	1.84	2.37	3.16	4.21	5.79	3.68	1.84	3.27	1.4326	2.0523
		LA	6.84	5.26	6.84	8.94	7.1	6.05	7.89	6.9886	1.1935	1.4243
	F	TTCL	15.77	16.3	20.33	15.77	16.82	18.05	11.74	16.3971	2.6097	6.8105
		TBCL	1.05	1.58	2.1	2.28	1.93	1.4	1.23	1.6529	0.4622	0.2136
		LA	10.51	12.44	0.18	10.69	1.75	12.09	12.79	8.6357	5.3277	28.3847
o	M	TTCL	14.21	24.46	31.83	29.99	23.15	12.63	11.05	21.0457	8.4642	71.642
		TBCL	5.67	10.14	8.04	8.04	8.3	4.36	8.3	7.55	1.9159	3.6706
		LA	4.74	6.31	4.47	5.79	6.31	4.74	4.47	5.2614	0.8441	0.7125
	F	TTCL	22.25	24.36	25.58	27.69	30.67	29.09	26.99	26.6614	2.8595	8.1765
		TBCL	5.71	5.53	7.46	6.94	6.24	6.94	7.99	6.6871	0.9052	0.8194
		LA	11.74	3.5	11.92	11.92	12.97	11.04	8.94	10.29	3.2426	10.5146
u/	M	TTCL	15.26	24.73	26.04	28.94	26.04	24.73	16.84	23.2257	5.1194	26.2082
		TBCL	4.74	4.74	3.16	4.74	3.95	4.74	3.16	4.1757	0.7514	0.5647
		LA	3.68	1.84	2.1	3.16	2.63	4.47	6.05	3.4186	1.4729	2.1696
	F	TTCL	18.92	21.55	28.04	29.61	31.19	28.56	25.06	26.1329	4.4976	20.2287
		TBCL	3.68	2.45	2.98	1.93	8.41	2.8	3.86	3.73	2.169	4.7046
		LA	2.98	1.75	1.93	2.45	8.76	1.75	1.75	3.0529	2.5591	6.5489

**Figure 7:**
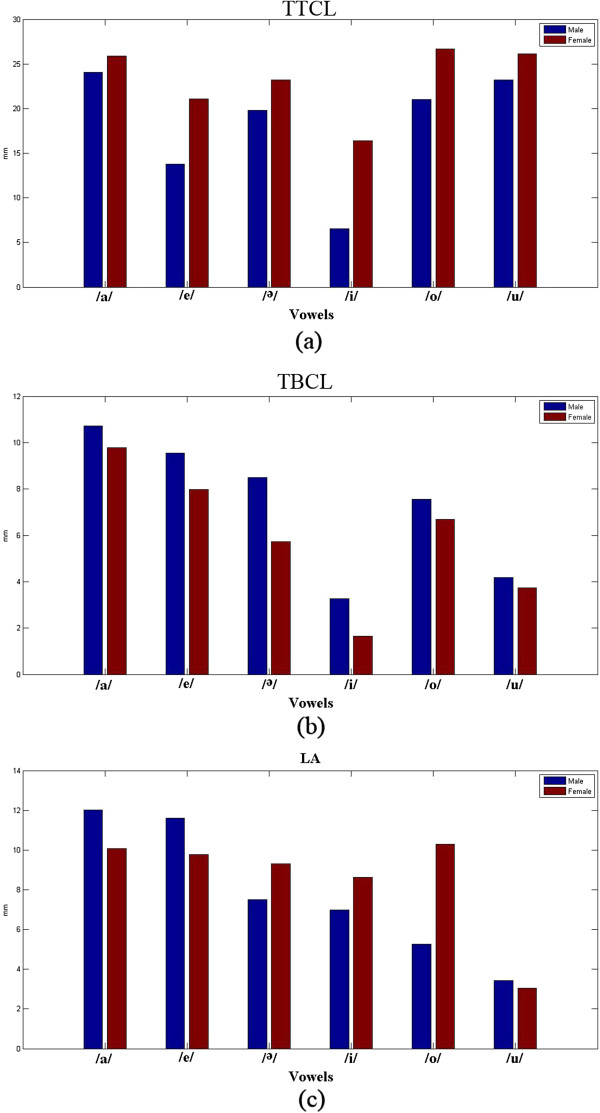
**The three articulatory parameters are (a) TTCL, (b) TBCL, and (c) LA.** The bar shows the average value of the articulatory parameters of each vowel, measured from the seven consecutive MR frames.

As expected from the standard IPA table, the TTCL and TBCL can provide information on the tongue position in the mouth given the shape of the palate [61]. For the vowels /i/ and /ә/, for which the tongue is positioned in front of the mouth (close to the teeth) during production, TTCL is lowest. The same holds for the TBCL. For the vowels /o/ and /u/, the tongue moves to the back of the oral cavity, which results in the highest amount of TTCL because the tongue tip is positioned in the middle of the oral cavity. Consequently, it has the largest distance to the palate. Figure 7 illustrates the value of the measured articulatory parameters TTCL, TBCL, and LA of the male and female subjects for the different Malay vowels.

## Discussion

As Figure 7(a) shows, the TTCL values of vowels /i/ and /ә/ are the lowest compared to those of the other vowels because they are front vowels and the TTCL parameter must be lower for the back vowels. Conversely, the back vowels /o/ and /a/ have the highest TTCL.In addition, Figure 7(b) presents the TBCL, which represents the height of the tongue in the articulation of different vowels. Given that /i/ is a high vowel, the value of the TBCL is at its lowest, while the vowel /a/, which is a low vowel, has the highest TBCL. Moreover, Figure 7(c) presents the LA value, which represents the lip aperture for the different vowels. The highest LA value is generated for vowel /a/ while the lowest is observed for vowel /u/. This result is attributed to the requirements in which the lips should be completely open when the vowel /a/ is articulated, but should be closer together when the vowel /u/ is produced.

**Figure 8:**
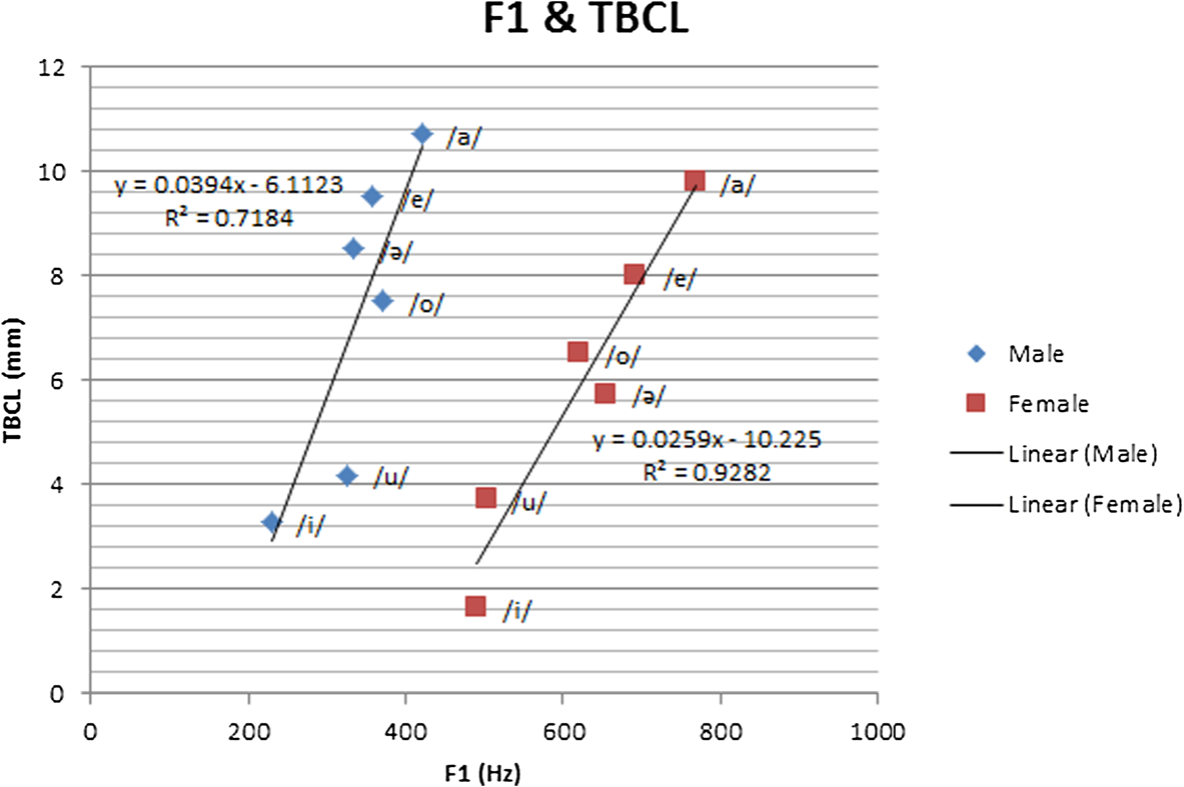
Correlation between F1 and TBCL.

**Figure 9:**
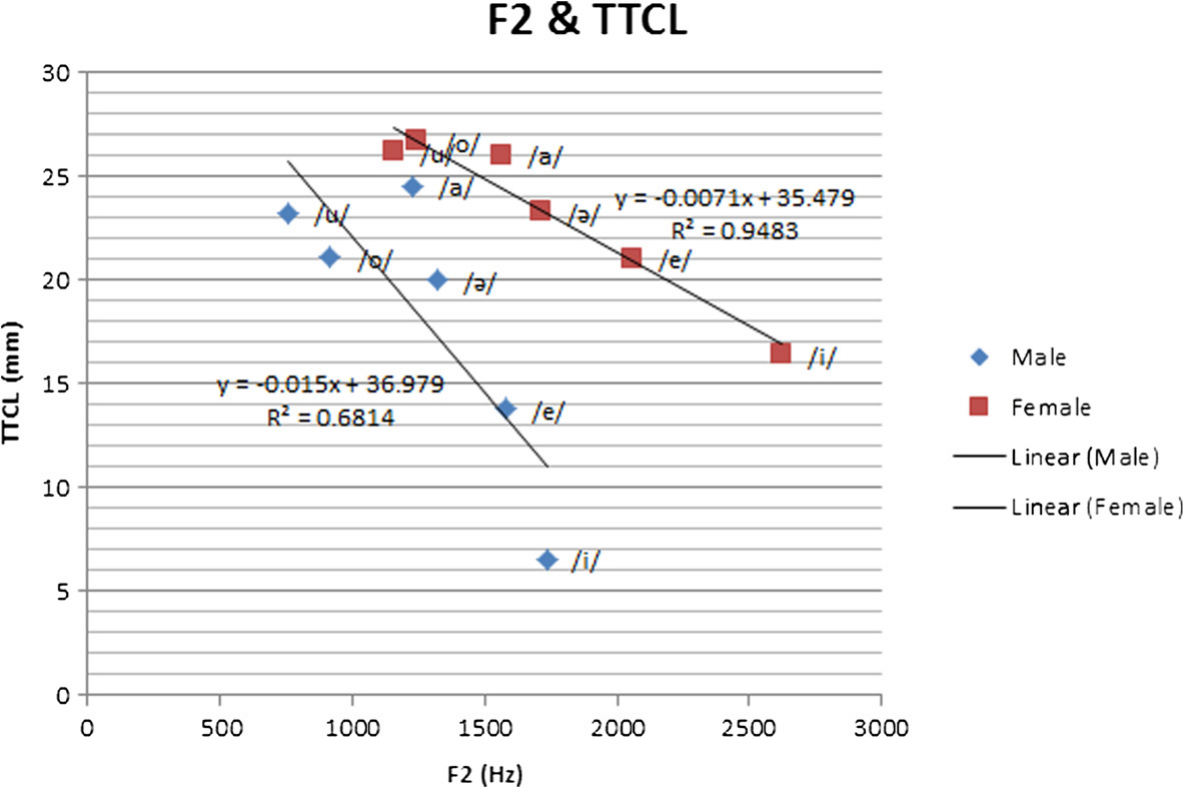
Correlation between F2 and TTCL.

Among the most significant parameters in speech analysis are formant frequencies, which have a crucial function in speech diagnosis and therapy applications. The relationship between the articulatory parameters obtained using MRI and formant frequencies has been studied [62]. The first formant frequency (F1) corresponds to vowel openness (vowel height). TBCL represents the height of the tongue. Thus, the TBCL value is related to F1. Our hypothesis on the direct positive correlation between F1 and TBCL is supported by the experimental results and the subjects’ formant frequencies, which are extracted from their voices. This hypothesis is also supported by Figure 8. The second formant frequency (F2) corresponds to the front vowels. The back vowels have low F2 frequencies, whereas the front vowels have high F2 frequencies. The back vowels have a high TTCL, whereas the front vowels have a low TTCL. Thus, we hypothesize that TTCL has a negative or indirect correlation with F2. This hypothesis is supported by the values of the subjects’ second formant frequencies, which are extracted from their voices. Figure 9 also shows the correlation between TTCL and F2, as well as that between TBCL and F1. The detail of the first and second formant frequencies are shown in Table 4.

**Table 4:** Formant Frequency values with STD

**Vowel**	**F1 male**	**F1 female**	**F2 male**	**F2 female**
a	420.48 ± 13.57	767.95 ± 12.25	1231.05 ± 25.17	1562.41 ± 27.54
e	358.12 ± 6.03	692.99 ± 7.03	1578.04 ± 24.33	2059.27 ± 22.54
ә	333.12 ± 2.72	654.43 ± 2.34	1324.39 ± 50.29	1711.98 ± 48.65
i	228.99 ± 0.60	490.61 ± 0.04	1738.69 ± 4.63	2624.81 ± 4.21
o	371.59 ± 5.26	620.58 ± 5.65	918.09 ± 2.03	1245.83 ± 2.38
u	326.49 ± 5.21	502.72 ± 4.86	754.98 ± 25.44	1157.55 ± 23.76

## Conclusion

In the study of speech articulation, MRI imaging yields helpful and precise information on the shape of articulators, as well as their position during speech production. Moreover, their dynamics can be appropriately investigated for the study of their temporal functions during articulation. However, the movement of articulators is an issue that demands higher temporal imaging resolution for a more accurate quantification. In this study, a proposed approach for this problem has been examined based on an image processing technique that uses active contours. After applying preprocessing methods to the MR images, we obtained the initial points for the active contours. Afterwards, the active contour was applied to the MRI frames. Consequently, the tongue contour was appropriately traced for the study of speech articulation parameters.

In the experiments, six Malay vowels were produced by the male and female subjects, and the articulatory parameters were measured using the proposed algorithm. The specific tongue shape and position for all the six Malay vowels were also obtained. The experiments demonstrated the correlations between acoustic speech and articulatory parameters. Specifically, the first formant frequency (F1) was positively correlated to TBCL, whereas the second formant frequency (F2) was negatively correlated to TTCL. The observations during this study can be helpful for researches regarding speech synthesis techniques. Furthermore, it can improve understanding of speech articulation in Malay language which can be useful for clinical usages of diagnosis of speech disorders and speech rehabilitation procedures.

## Competing interests

The authors declare that they have no competing interests.

## Authors’ contributions

AZ: drafted the manuscript, as a main and corresponding author and did the experiments and analyzed the acoustic parameters data and compared with other author’s results of analysis. SMM: participated in the data analysis of articulatory parameters and MRI images processing and drafted the manuscript. HNT: conceived of the study and participated in its design and coordination as a main supervisor of the project and helped to draft the manuscript. SIB: participated in the design of the study especially for the MRI experiments and helped to draft the manuscript. KHN: participated in the design of the study and helped to draft the manuscript. MB: participated in the design of the study and helped to draft the manuscript as well as helped to design MRI image processing procedure. MAJ: participated in the design of the study and helped to draft the manuscript. All authors read and approved the final manuscript.
